# Duplication and Diversification of *REPLUMLESS* – A Case Study in the Papaveraceae

**DOI:** 10.3389/fpls.2018.01833

**Published:** 2018-12-12

**Authors:** Cecilia Zumajo-Cardona, Natalia Pabón-Mora, Barbara A. Ambrose

**Affiliations:** ^1^New York Botanical Garden, Bronx, NY, United States; ^2^The Graduate Center, City University of New York, New York, NY, United States; ^3^Instituto de Biología, Universidad de Antioquia, Medellín, Colombia

**Keywords:** basal eudicots, *Bocconia frutescens*, fruit development, Papaveraceae, *Papaver somniferum*, *REPLUMLESS*, replum

## Abstract

There is a vast amount of fruit morphological diversity in terms of their texture, the number of carpels, if those carpels are fused or not and how fruits open to disperse the seeds. *Arabidopsis thaliana*, a model eudicot, has a dry bicarpellate silique, when the fruit matures, the two valves fall apart through the dehiscence zone leaving the seeds attached to the remaining medial tissue, called the replum. Proper replum development in *A. thaliana* is mediated by REPLUMLESS (RPL), a TALE Homeodomain protein. RPL represses the valve margin genetic program and the downstream dehiscence zone formation in the medial tissue of the siliques and RPL orthologs have conserved roles across the Brassicaceae eudicots. A *RPL* homolog, *qSH1*, has been studied in rice, a monocot, and plays a role in fruit shedding making it difficult to predict functional evolution of this gene lineage across angiosperms. Although *RPL* orthologs have been identified across all angiosperms, expression and functional analyses are scarce. In order to fill the phylogenetic gap between the Brassicaceae and monocots we have characterized the expression patterns of *RPL* homologs in two poppies with different fruit types, *Bocconia frutescens* with operculate valvate dehiscence and a persistent medial tissue, similar to a replum, and *Papaver somniferum*, a poppy with persistent medial tissue in between the multicarpellate gynoecia. We found that *RPL* homologs in Papaveraceae have broad expression patterns during plant development; in the shoot apical meristem, during flowering transition and in many floral organs, especially the carpels. These patterns are similar to those of *RPL* in *A. thaliana*. However, our results suggest that *RPL* does not have conserved roles in the maintenance of medial persistent tissues of fruits but may be involved with establishing the putative dehiscence zone in dry poppy fruits.

## Introduction

The *Arabidopsis* *thaliana* (Arabidopsis) gynoecium is composed of two congenitally fused carpels, which after fertilization develop into a dry dehiscent fruit, known as a silique. This fruit is formed by two valves, which during dehiscence, separate from the replum by the tension created against the rigid lignified layer (Roeder and Yanofsky, 2006). The replum was originally described as the tissue where the seeds remain attached after the two valves fall apart but is now described as only the outer or abaxial portion and does not include the inner septum (Ferrandiz et al., 1999; Alvarez and Smyth, 2002). The gene regulatory network involved in Arabidopsis fruit development has been extensively described (Ferrandiz et al., 1999; Ripoll et al., 2011; Reyes-Olalde et al., 2013; Chávez-Montes et al., 2015). One of the genes involved in proper replum development is *REPLUMLESS* (RPL; Roeder et al., 2003). RPL belongs to the TALE class of Homeodomain proteins with a TALE motif within the triple helix of the Homeodomain (HD) but are characterized from other TALE-HD proteins by a ZIBEL motif (Bürglin, 1997; Becker et al., 2002; Kumar et al., 2007; Mukherjee et al., 2009). Comprehensive analyses of RPL related sequences have found that these TALE proteins are closely related to the BELL proteins (Mukherjee et al., 2009), therefore they are also called BELL-like Homeodomain proteins (BLH; Chan et al., 1998; Becker et al., 2002; Roeder et al., 2003; Hake et al., 2004). *RPL* has broad expression patterns during *A. thaliana* development, with the highest expression levels detected in the stems, and in the replum beginning early in floral development (Byrne et al., 2003; Roeder et al., 2003; Dinneny et al., 2005; Kanrar et al., 2006; Yu et al., 2009; Avino et al., 2012; Khan et al., 2012, 2015; Chung et al., 2013; Arnaud and Pautot, 2014; Andrés et al., 2015). The *rpl* mutant, as the name suggests is defective in replum development in the fruit, however, mutants of this gene also have vegetative defects (Roeder et al., 2003). *rpl* (also known as *pennywise*, *bellringer*, and *vaamana*) shows partial loss of apical dominance, shorter plants and defects in phyllotaxy (Byrne et al., 2003; Roeder et al., 2003; Smith and Hake, 2003; Bhatt et al., 2004). RPL maintains meristem identity by maintaining cell proliferation and repressing lateral organ boundary genes such as *BLADE-ON-PETIOLE1/2* (Khan et al., 2015). Moreover, during late fruit development RPL is restricted to the replum and negatively regulates *SHATERPROOF*, a MADS-box gene involved in the specification of the dehiscence zone (Roeder et al., 2003; Kramer et al., 2004; Fourquin and Ferrandiz, 2012). Meanwhile, RPL is also directly repressed by APETALA2 (AP2), a protein that belongs to the AP2/ERF transcription factor family which is upstream of the entire fruit developmental network (Ripoll et al., 2011). RPL restricts valve and valve margin development and therefore is indirectly involved in proper replum formation (Alonso-Cantabrana et al., 2007).

*REPLUMLESS* orthologs have been identified across all angiosperms and are the result of a duplication event before angiosperm diversification that also gave rise to its sister clade *POUND FOOLISH* (*PNF*; Pabón-Mora et al., 2014). However, expression and functional studies are scarce outside Arabidopsis. In *Lepidium* species, also in the Brassicaceae, *RPL* expression is found only in leaves, at the tip of the inflorescence meristem and in developing flowers while absent from older flowers or in fruits (Mühlhausen et al., 2013). In *Oryza sativa* (rice), *RPL* appears to be one of the genes involved in its domestication. At maturity, wild rice disperses the fruit with the seed inside to guarantee propagation while, in domesticated rice the fruit remains attached to the plant to make harvest easy and increase production (Lin et al., 2007; Arnaud et al., 2011; Meyer and Purugganan, 2013). The domesticated rice phenotype is the result of a mutation in the promoter of *Seed Shattering in Chromosome 1* (*qSH1*, the *RPL* homolog in rice) which controls the formation of the abscission layer at the base of the sterile bract (Konishi et al., 2006; Lin et al., 2007; Gasser and Simon, 2011). Available functional data suggest that *RPL* genes play different roles in Brassicaceae and monocots during flower and fruit development thus, comparative data is needed in order to assess their expression and functional evolution across angiosperms.

**FIGURE 1:**
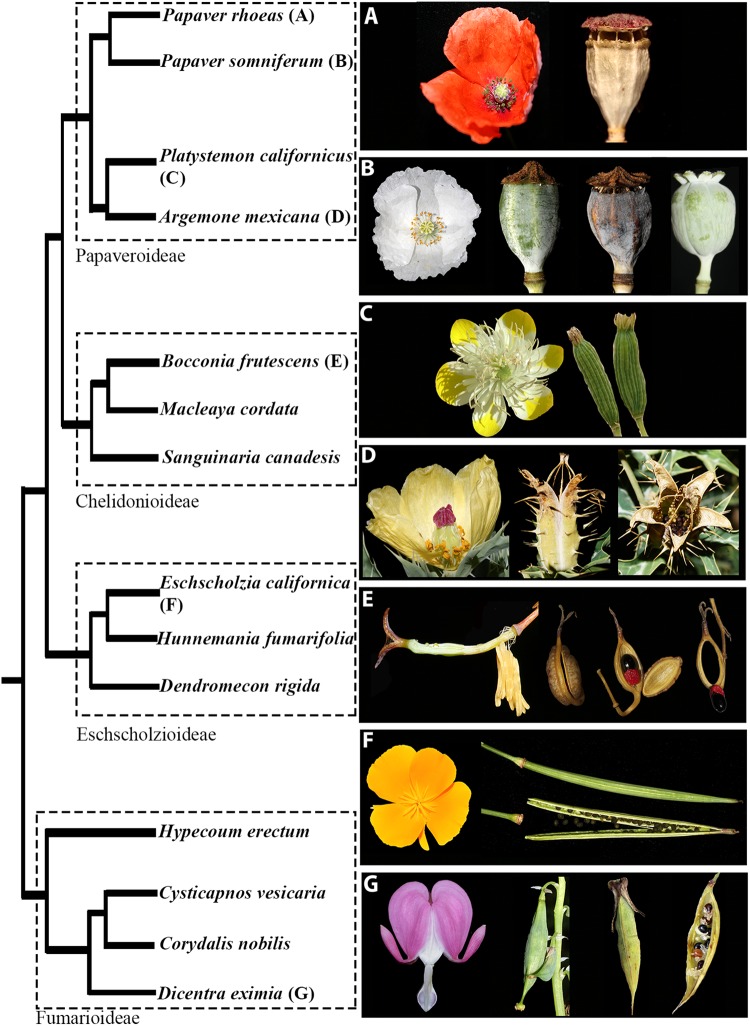
The relationships of selected Papaveraceae with representatives from the four subfamilies: Papaveroideae, Chelidonioideae, Eschscholzioideae, and Fumarioideae. Pictures of representative species illustrate the flower and fruit morphological diversity within the family. Poricidal capsules in **(A)** *Papaver rhoeas* and **(B)** *Papaver somniferum*. Note that *P. somniferum* cv. Persian White (to the right in **B**) lacks pores. Capsules that separate into mericarps in **(C)** *Platystemon californicus.* Longitudinally dehiscent capsules in, **(D)** *Argemone mexicana*, **(F)** *Eschscholzia californica* and **(G)** *Dicentra eximia*. Capsules with opercular dehiscence in **(E)** *Bocconia frutescens.*

Here, we investigate the expression patterns of *RPL* homologs in the Papaveraceae, as they are members of the basal eudicots and exhibit different strategies for seed dispersal. Using fruit diversity within this family is key to understanding the mechanisms involved in defining the medial zone in different dry dehiscent fruit morphologies. Fruit diversity in Papaveraceae includes dry dehiscent fruits with poricidal capsules with pores coinciding with locule number as in *Papaver* (Sárkány and Szalay, 1964; Roth, 1977; Gunn, 1980; Kapoor, 1995) (Figures 1A,B), schizocarps as in *Platystemon* (Figure 1C), pores that extend basipetally leaving “baskets” full of seeds, as in *Argemone* (Figure 1D), fruits with complete longitudinal dehiscence with a remaining septum as in *Eschscholzia* (Becker et al., 2005) (Figure 1F) or in *Dicentra* (Figure 1G) and fruits with opercular dehiscence where the two valves, derived from the two carpels, fall apart from a remaining ring-like commissural tissue where the seed remains attached as in *Bocconia* (Zumajo-Cardona et al., 2017) (Figure 1E). The type of fruit dehiscence found in *Bocconia* resembles the dehiscence of the Arabidopsis fruit.

The fact that some species within Papaveraceae (i.e., *B. frutescens*) resemble the Arabidopsis silique, will allow us to understand if the same genetic network has been co-opted at different evolutionary points for similar types of fruit. In addition, the Papaveraceae belong to the order Ranunculales, which form a well-supported clade placed as the sister group to the core eudicots (Kim et al., 2004; Angiosperm Phylogeny Group, 2016) and therefore, occupy a key phylogenetic position outside of the well studied core eudicots and monocots (Drea et al., 2007). Here, we describe the expression patterns of *RPL* homologs in two poppies: *P. somniferum* and *B. frutescens* in order to assess the putative role of *RPL* genes in different fruit types in basal eudicots. This in turn will help us understand the shifts that the *RPL* gene lineage has undergone during angiosperm evolution.

## Materials and Methods

### Gene Homolog Searches and Phylogenetic Analysis

We performed BLASTN targeted searches in previously assembled transcriptomes of *Bocconia frutescens* (Papaveraceae) (Arango-Ocampo et al., 2016; Zumajo-Cardona et al., 2017), using as query sequences those previously reported for other Papaveraceae *RPL* homologs [i.e., *Papaver somniferum RPL* (KKCW-2001151, OneKP) and *Sanguinaria canadensis RPL* (XHKT-2009137, OneKP; Pabón-Mora et al., 2014)] as well as the *Arabidopsis thaliana* sequence (AT5G02030).

To assess the phylogenetic position of the *Bocconia frutescens* homologs, we included *BofrRPL1*, *2* and *3* in a matrix consisting of selected *RPL* homologs from all major plant groups, based on the sampling done by Pabón-Mora et al. (2014). Additionally, we extended the sampling particularly in basal eudicots from the plant transcriptome repositories of the oneKP database^1^ and PhytoMetaSyn^2^.

A total of 117 sequences from all major angiosperm groups were compiled and edited manually to exclusively keep the open reading frame for all transcripts using AliView (Larsson, 2014). Nucleotide sequences were subsequently aligned using the online version of MAFFT^3^ (Katoh et al., 2002) with a gap open penalty of 3.0, offset value of 0.5, and all other default settings. The resulting alignment was refined manually using AliView. Maximum Likelihood (ML) phylogenetic analysis using the complete nucleotide alignment of all homologs was performed through the CIPRES Science Gateway (Miller et al., 2010) with RaxML-HPC2 BlackBox (Stamatakis et al., 2008). Three *PNF* sequences from *A. thaliana*, *A. lyrata* and *C. rubella* (Brassicaceae) were used as the outgroup (Supplementary Table S1). Trees were observed and edited using FigTree v 1.4.3^4^. Newly isolated sequences from our *Aristolochia fimbriata* and *B. frutescens* transcriptomes are available under GenBank numbers MK057522 – MK057526. To detect conserved motifs, 28 complete protein sequences from Ranunculales were selected. The sequences were permanently translated and uploaded to the online MEME suite^5^ (Bailey et al., 2006) and run using all the default options set to find 20 motifs.

### Carpel and Fruit Morphology and Anatomy

*P. somniferum* seeds were germinated in a growth chamber under controlled conditions with 15 h of light and a relative humidity of 60%. After germination, plants were grown to maturity under the same conditions. Flowers in preanthesis, anthesis and fruits at several developmental stages were collected and fixed in formaldehyde-acetic acid–ethanol (FAA; 3.7% formaldehyde: 5% glacial acetic acid: 50% ethanol). *B. frutescens* was collected in the field (voucher: Colombia, Antioquia, Medellín, Las Palmas, Envigado, sobre la via principal, Km 12 retorno No. 10. May 2015, *C. Zumajo*-*Cardona* and *N. Pabón*-*Mora* 03) and immediately fixed in FAA. The material was dehydrated through an alcohol-histochoice series and embedded in Paraplast X-tra (Fisher Healthcare, Houston, TX, United States). The samples were sectioned at 10 μm with a MICROM HM355 (Fisher Scientific, Pittsburgh, PA, United States) rotary microtome. Sections were stained with Johansen’s safranin, to identify lignification and presence of cuticle, and 0.5% Astra Blue (Kraus et al., 1998) and mounted in Permount (Fisher Scientific, Pittsburgh, PA, United States). Sections were viewed on a Zeiss optical microscope and digitally photographed with a Nikon DXM1200C digital camera and ACT-1 software. In addition, comparative morphological analyses between the Papaveraceae fruits was done based on fresh material as shown in Figure 1.

### *In situ* Hybridization Expression Analyses

*P. somniferum* and *B. frutescens* vegetative apices, inflorescences, floral buds and fruits at different developmental stages were collected, fixed in cold FAA and processed similarly as described above for anatomy samples. Paraplast X-tra embedded samples were maintained at 4°C until use. Samples were sectioned with a rotary microtome (Microm HM3555) at 8 μm. DNA templates for RNA probe synthesis were obtained by PCR amplification of 300–370 bp fragments. To ensure specificity, the probe templates were designed to amplify the 3′ sequence flanking the Homeodomain (Supplementary Table S2 and Supplementary Figure S1). Fragments were cleaned using QIAquick PCR purification Kit (Qiagen, Valencia, CA, United States). Digoxigenin labeled RNA probes were prepared using T7 RNA polymerase (Roche, Switzerland), RNAse inhibitor RNasin (New England Biolabs, Ipswich, MA, United States) and RNA labeling-mix (Roche, Switzerland) according to the manufacturer’s protocol. RNA *in situ* hybridization was performed according to Ambrose et al. (2000). There are a minimum of three replicates and up to nine for each probe and each developmental stage within each species. *In situ* hybridized sections were subsequently dehydrated and permanently mounted in Permount (Fisher, Waltham, MA, United States). All sections were digitally photographed using a Zeiss Axioplan microscope equipped with a Nikon DXM1200C digital camera.

## Results

### *REPLUMLESS* Gene Evolution

**FIGURE 2:**
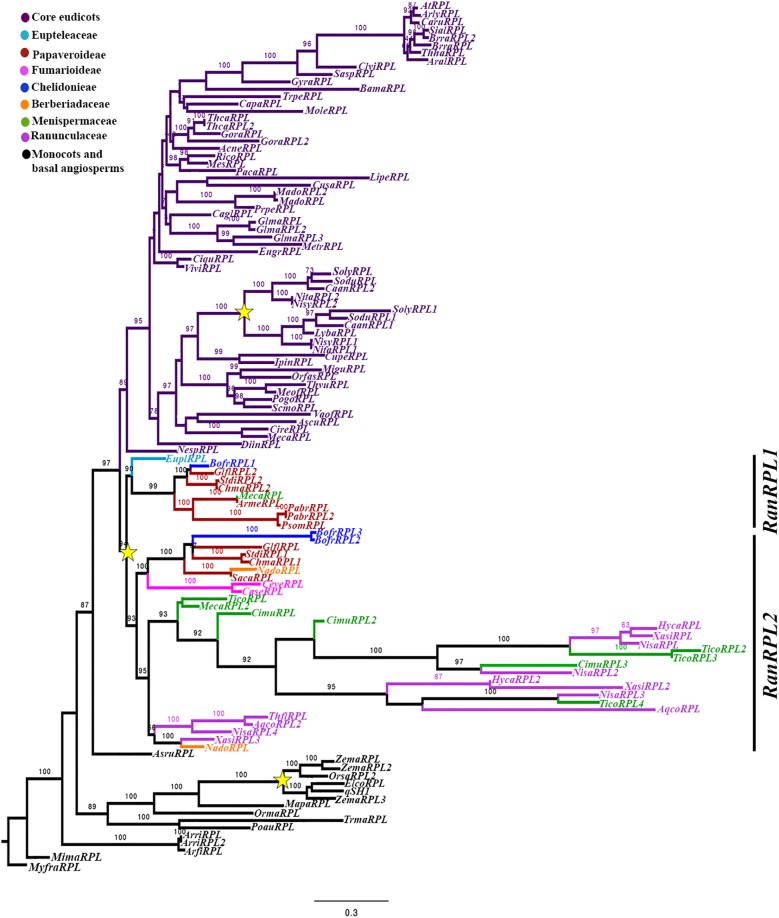
Maximum Likelihood analysis for the *RPL* gene lineage in angiosperms with expanded sampling in basal eudicots using three *PNF* sequences from *A. thaliana*, *A. lyrata* and *C. rubella* (Brassicaceae) as an outgroup. Bootstrap values, larger than 60 are shown at nodes. Two clades were identified for Ranunculales (basal eudicots), *RanRPL1* and *RanRPL2*. Yellow stars indicate two large-scale duplication events, the first one in the Poaceae (Pabón-Mora et al., 2014) and the second one previous to the diversification of the Solanaceae (Ortíz-Ramírez et al., 2018).

To reconstruct the *RPL* gene lineage evolution, we used the complete coding sequence of 117 homologs from all major angiosperm groups (Supplementary Table S1). The sister clade *POUNDFOOLISH* (Pabón-Mora et al., 2014) was used as the outgroup. Maximum Likelihood analysis recovered independent duplication events before the diversification of Poaceae (BS = 100) and Solanaceae (BS = 100) as has been previously reported (Figure 2) (Pabón-Mora et al., 2014; Ortíz-Ramírez et al., 2018). Here, we recovered an additional duplication likely predating the diversification of Ranunculales (basal eudicots). We have named the two resulting clades as *RanRPL1* and *RanRPL2* (Figure 2). However, it is unclear if the duplication occurred before or after the radiation of Eupteleaceae, as there is a single *RPL* sequence from *Euptelea pleiosperma* in the *RanRPL1* clade. In addition, the *RanRPL1* clade includes Papaveraceae sequences and a single sequence of Menispermaceae (BS = 90) (Figure 2). The *RanRPL2* clade (BS = 93) includes Papaveraceae, Berberidaceae, Menispermaceae, and Ranunculaceae sequences. Multiple *RPL* sequences were identified for *Tinospora cordifolia* (Menispermaceae), *Hydrastis canadensis*, *Nigella sativa* and *Xanthorhiza simplicissima* (Ranunculaceae; Figure 2) but the topology does not allow us to determine if these sequences are the result of an additional duplication event predating Menispermaceae and Ranunculaceae. Finally, taxon-specific duplications in the *RPL* clade have occurred multiple times, usually associated with recent whole genome duplication (WGD) events as in the case of *Bocconia frutescens, Glycine max, Malus domestica*, *Papaver bracteatum, Theobroma cacao*, and *Tinospora cordifolia* (Figure 2) (Davie, 1935; Sugiura, 1940; Schmutz et al., 2010; Argout et al., 2011; Jain and Prasad, 2014; Panchy et al., 2016).

**FIGURE 3:**
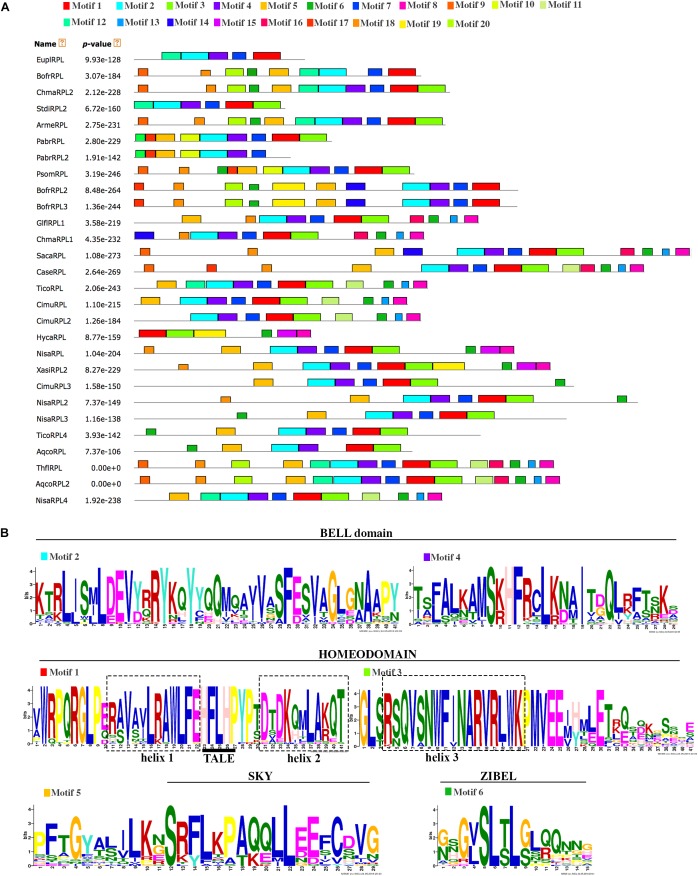
**(A)** Conserved motifs across the Ranunculales RPL proteins identified through a MEME analysis. Each conserved motif is represented by a colored box numbered at the top. Gray lines represent unique sequences. **(B)** Sequences of the conserved motifs previously identified for the RPL proteins such as the BELL domain, Homeodomain, SKY motif and ZIBEL motif. The Three Amino acid Loop Extension (TALE) in the Homeodomain is underlined.

All of the RPL proteins included in the phylogenetic analysis contained the Homeodomain, the BELL domain as well as the SKY and ZIBEL motifs already characterized as highly conserved across RPL homologs (Figure 3) (Mukherjee et al., 2009; Pabón-Mora et al., 2014). The ZIBEL motif can be found before the SKY motif (i.e., BofrRPL1, 2, and 3, ChmaRPL2, PsomRPL), or after the Homeodomain (i.e., GlflRPL1, SacaRPL, CaseRPL; Figure 3). Additionally, we identified 14 new motifs that are highly conserved within basal eudicots (Supplementary Figure S2). Nine of these motifs (Motifs 9-14, 16, 17, and 20) are lacking in the Ranunculaceae and Menispermaceae protein sequences: HycaRPL, NisaRPL1/2/3, XasiRPL2, CimuRPL3, TicoRPL4, and AqcoRPL, (Figure 3). These differences may explain the long branch formed in the *RanRPL2* clade and suggest a relaxed negative selection (Figure 2). Interestingly, the MEME analysis found that located before the BELL domain is motif 10, FVDQDC/SCLMESSEDRLDCSDDQDEHHHWR (Supplementary Figure S2), which is rich in negatively charged and hydrophobic amino acids and is exclusively found in *Papaver* sequences (Figure 3).

### *Bocconia frutescens* Flower and Fruit Development

**FIGURE 4:**
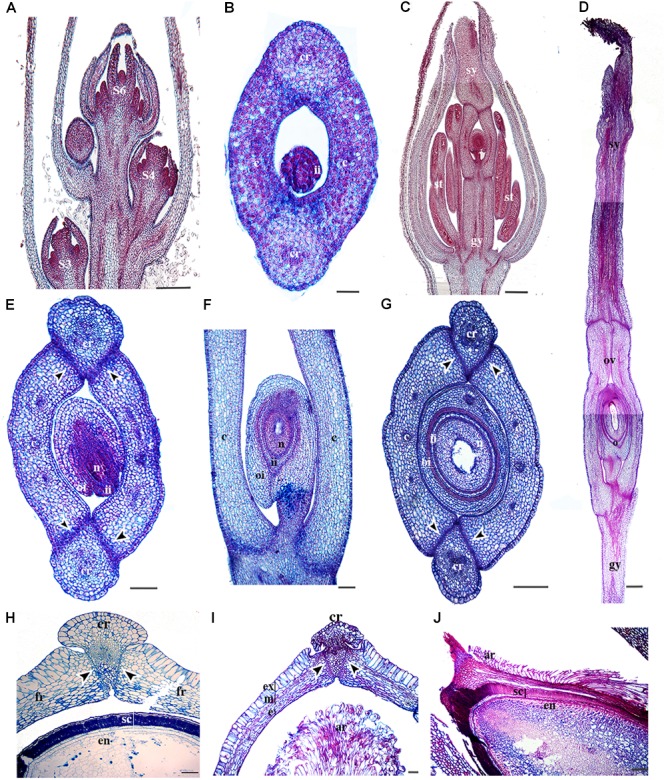
Anatomy of *B. frutescens* flowers and fruits. **(A)** Longitudinal section of an inflorescence with flowers at different stages (S): S3, S4, and S6. Stages follow Zumajo-Cardona et al. (2017). **(B)** Cross-section of a carpel at S6. **(C)** Longitudinal section of a flower at S8. **(D)** Longitudinal section of the gynoecium at S9, showing the three main proximo-distal zones: gynophore, ovary, and style with the two stigmas at the apex. **(E)** Cross-section of a flower at S8. **(F)** Close-up longitudinal section of an ovary at S9. **(G)** Cross-section of the ovary at S9. **(H)** Cross-section of a young fruit at S10 and the seed. **(I)** Cross-section of a fruit at S11 showing fruit layers: exocarp, mesocarp and endocarp. **(J)** Longitudinal section of the seed coat and aril. *Black arrowheads* indicate the separation layer, ar, aril; b, bract; c, carpel; cr, commissural ring (persistent medial tissue); e, endocarp; en, endosperm; ex, exocarp; fr, fruit wall; gy, gynophore; ii, inner integument; m, mesocarp; n, nucleus; o, ovule; oi, outer integument; ov, ovary; s, sepal; sc, seed coat; se, seed; st, stamen; sy, style; v, valve. Scale bars: 100 μm **(B,I–J)**, 200 μm **(D)**, 250 μm **(H)**, 0.2 mm **(E)**, 0.5 mm **(A,C,F,G)**.

To better hypothesize the role of *RPL* homologs in basal eudicots, we examined their expression throughout the 11 flower and fruit developmental stages of *Bocconia frutescens* (Papaveraceae) that have been previously defined (Zumajo-Cardona et al., 2017). In addition, we provide anatomical details during later stages of fruit development. Briefly, the determinate inflorescences of *Bocconia frutescens* L., has numerous apetalous flowers that develop basipetally (Figure 4A). All flowers are composed of two sepals, a single whorl of homeotic stamens replacing petals, two- three whorls of true stamens and a bicarpellate gynoecium (Figures 1E, 4A) (Arango-Ocampo et al., 2016). The two sepal primordia initiate at stage 2, followed by the initiation of the first whorl of homeotic stamens during stage 3 (Figure 4A). At stage 4, all the staminal whorls are formed, usually 2 to 3 (Figure 4A). The two carpels start developing during stage 5, so that by stage 6 the two carpels overtop the single ovule (Figure 4A). During stage 7, the apical region of the carpel differentiates forming the style and stigmas with each carpel composed of 8–10 cell layers. At stage 7, the carpels meet in a commissural ring-like tissue and four main vascular traces are apparent; one in each carpel and one in each of the commissural rings and the cells of the commissural rings are not as densely stained. Also during stage 7, the inner integument of the ovule begins to develop (Figure 4B). During floral developmental stages 8–9, three main proximo-distal zones differentiate: the gynophore, the ovary and the style (Figures 4C,D). The style rapidly extends and is topped with two massive papillate stigmas (Figure 4D). At stage 8, the ovary wall is composed of 12 cell layers and a 4–5 cell separation layer, between the carpels and the commissural ring, becomes distinct (Figure 4C). Also at stage 8, the ovule is developing with the inner integument covering the nucellus while the outer integument is still elongating (Figure 4E). At stage 9 (Figures 4D,F,G), the two integuments completely cover the single anatropous ovule, forming what is going to be the seed coat; the ovary walls are formed by 12–15 cell layers of parenchymatous tissue (Figures 4D,F,G), with three vascular bundles (one central and two lateral) vascular bundles in each carpel and a vascular bundle in the commissural ring, as in stage 8 (Figure 4E). As detailed fruit developmental stages have not been described, here, we provide cross sections of the fruits at distinct stages (Figures 4H–J). After pollination, the two carpels become the two valves. The number of vascular bundles in the valves and the commissural ring remains the same (Figure 4H). During the first stage of fruit development, stage 10 (Figure 4H), the 12–15 cells forming the pericarp become more compressed and can be further distinguished into the exocarp with a single cell layer of rectangular tightly packed cells, covered by a thin cuticle, the mesocarp formed by larger isodiametric parenchymatous cells with three vascular bundles, and the endocarp formed by smaller cells than the exocarp. The separation layers between the valves and the persistent commissural ring are composed of 2–3 cell layers of densely stained cells. The commissural ring is wedge-shaped, formed by parenchymatous tissue with one vascular bundle (Figure 4H). Also during this stage the seed is fed by a vascular bundle and it is possible to differentiate the endosperm and the seed coat which develops from the two integuments, the three inner layers of the seed coat arise from the inner integument and the 6–8 outer layers from the outer integument (Figures 4F,G,I). During stage 11 of fruit development, (Figures 4I,J), the pericarp becomes differentiated into an exocarp of radially elongated and enlarged cells, a mesocarp formed by 6–7 of tangentially elongated cells and, an endocarp with 3–4 layers of very small flat cells (Figure 4I). The epidermal cells of the commissural ring are different from the exocarp of the valves; cells in the commissural ring are smaller (Figure 4I). When the fruit ripens as stage 12 the two valves fall off leaving only the seed attached to the commissural ring (see *Bocconia* fruit, Figure 1E). The base of the seed is covered with a red aril that originates from the funiculus after fertilization; during the ripening of the seed it does not form part of the seed coat as it develops a distinctive coloring and texture (Figures 4I,J). The aril in *Bocconia* serves to attract birds since the brilliant red color of it contrasts with the black testa (see *Bocconia* fruit, Figure 1E). Arils occur on seeds of many tropical species. Interestingly, *Bocconia* is the only neo-tropical genus from the Papaveraceae and the only one within the family that has an aril.

### Expression of *RPL Bocconia frutescens* Homologs (*BofrRPL1/2/3*)

**FIGURE 5:**
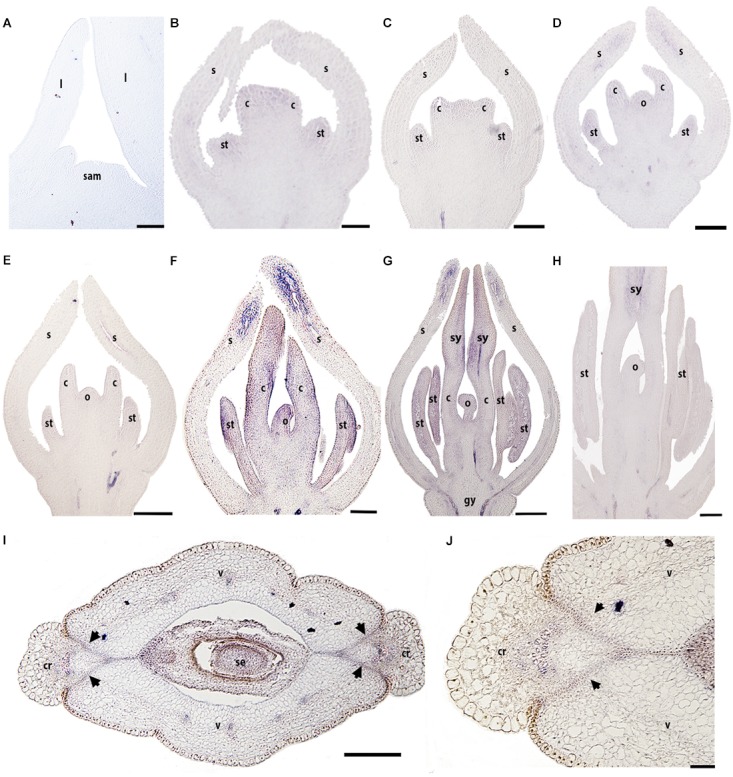
Expression of *BofrRPL1* by *in situ* hybridization in longitudinal **(A–H)** and cross-sections **(I,J)** of developing shoots, flowers, and fruits. **(A)** *BofrRPL1* expression is not detected in the shoot apical meristem, **(B)** floral bud in stage 3, **(C–E)** or floral stages 4-6. **(F)** *BofrRPL1* expression is first detected at floral stage 7 in stamens, carpels, and ovule. **(G,H**) At stage 8 *BofrRPL1* is restricted to the region where the two stigmas fuse floral. **(I,J)** *BofrRPL1* expression becomes restricted during fruit development and is detected in the dehiscence zone. *Black arrows* indicate the dehiscence zones, b, bract; c, carpel; cr, commissural ring; gy, gynophore; l, leaf; o, ovule; s, sepal; sam, shoot apical meristem; se, seed; st, stamen; sy, style; v, valve. Scale bars: 50 μm **(A–C)** 100 μm **(D)**, 0.1 mm **(E–H)**, 0.2 mm **(I)**, 500 μm **(J)**.

We evaluated the expression patterns of the three *REPLUMLESS* paralogs (*BofrRPL1/2/3*) in *B. frutescens* with specific probes designed for each one (Supplementary Table S2 and Supplementary Figure S1). Our results show different expression patterns for the three *Bocconia frutescens RPL* homologs. *BofrRPL1* expression was not detected in the vegetative meristem, young leaves (Figure 5A) or during the initiation of floral organ primordia at stages 3–6 (Figures 5B–E). Low levels of *BofrRPL1* expression are detected at the sepal tips, specifically in the vascular traces during stages 4–5 (Figures 5D,E). Expression of *BofrRPL1* is stronger later in flower development, at stage 6 when the two carpels overtop the single ovule, where *BofrRPL1* is detected in the sepal tips, stamens, both in the anthers and the filaments, the adaxial side of the carpels and in the tip of the nucellus during ovule elongation prior to integument initiation (Figure 5F). During stages 7 and 8 when differentiation of the style and stigma occur, *BofrRPL1* expression is maintained in the sepal tips, the stamens, the adaxial region of each style and stigma toward the medial region where fusion will occur, and the ovule (Figures 5F,G). However, in the fully differentiated gynoecium, after syncarpy, *BofrRPL1* becomes restricted to the proximal region of the long styles specifically toward their adaxial epidermis where fusion has occurred (Figure 5H). At this stage, expression of *BofrRPL1* was also found in the vasculature at the base of the flower (Figures 5G,H). During the transition to fruit development, between stages 9–10, *BofrRPL1* is expressed in the 3–4 cell layers between the valves and the commissural ring that will form the dehiscence zone in the mature fruits (Figures 5I,J). *BofrRPL1* is also expressed in the vascular bundle that feeds the commissural ring as well as in the three vascular bundles found in each valve (Figures 5I,J).

**FIGURE 6:**
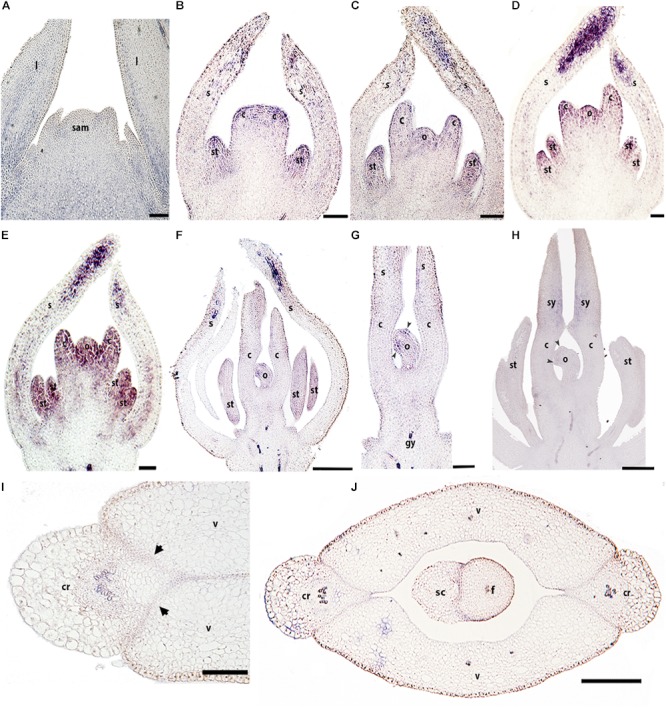
Expression analyses of *BofrRPL2* by *in situ* hybridization in longitudinal **(A–H)** and cross-sections **(I,J)** of developing shoots, flowers, and fruits. **(A)** *BofrRPL2* expression is detected in the adaxial side of leaf primordia and in more mature leaves. **(B–F)** *BofrRPL2* expression is detected in floral stages 3–7 in stamen and carpel primordia. **(C–E)** These expression patterns are maintained from stages 4 to 6, where *BofrRPL2* is also expressed in the ovule primordia. **(F)** At stage 7 the expression in the ovule is restricted to the initiation of the integuments. **(G)** *BofrRPL2* expression is only detected in the style and integument primordia during floral stages 8–9. **(I–J)** *BofrRPL2* expression is restricted to the dehiscence zone during fruit development. *Black arrowheads* indicate integument primordia, *Black arrows* indicate the dehiscence zones of the fruit, b, bract; c, carpel; cr, commissural ring; f, funiculus; gy, gynophore; l, leaf; o, ovule; s, sepal; sam, shoot apical meristem; sc, seed coat; se, seed; st, stamen; sy, style; v, valve. Scale bars: 50 μm **(A)**, 100 μm **(B–E)**, 0.1 mm **(H)**, 0.2 mm **(F,G,I,J)**.

Unlike *BofrRPL1*, *BofrRPL2* is detected in early vegetative and floral development. *BofrRPL2* is found during vegetative development on the adaxial side of the leaf primordia where it is maintained during leaf growth (Figure 6A). During early flower development, between stages 3–6, *BofrRPL2* is expressed in the sepals, particularly at their tips, in stamen and carpel primordia (Figure 6B), as well as in the ovule primordium (Figures 6C–E). During stages 7–9, the expression of *BofrRPL2* decreases dramatically, nevertheless it is still detected in the sepal tips, the anthers, the adaxial side of the carpels where the styles will fuse and the tips of the ovule where the two integuments will develop (Figures 6F–H). During fruit development at stages 10–11, *BofrRPL2* expression is found in cell layers between the valves and the commissural ring, the region that will correspond to the dehiscence zone during fruit ripening, as well as in the seed (Figures 6I,J).

**FIGURE 7:**
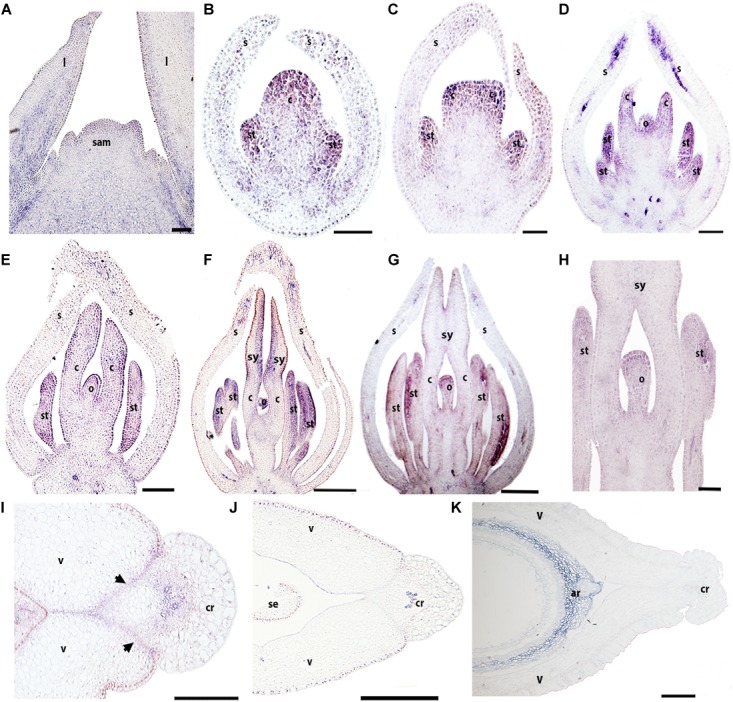
Expression of *BofrRPL3* by *in situ* hybridization of longitudinal **(A–H)** and cross-sections **(I–K)** of developing flowers and fruits. **(A)** *BofrRPL3* expression is first detected in the apex of the shoot apical meristem, in leaf primordia and in the adaxial side of more developed leaves. **(B–F)** *BofrRPL3* expression is detected in floral stages 2–7. **(B)** *BofrRPL3* expression is detected in the stamen and carpel primordia as they emerge and **(C)** as the two carpel primordia begin to elongate. **(D–H)** *BofrRPL3* expression persists in stamens, in carpels as they elongate and is also detected throughout ovule development up until floral stages 8–9. **(I)** *BofrRPL3* expression is detected in the gynoecium at S9 between the valves and the comissural ring. **(J)** *BofrRPL3* expression becomes restricted to the epidermis during fruit development. **(K)** *BofrRPL3* expression is restricted to the aril during seed development. *Black arrows* indicate the dehiscence zones. ar, aril; b, bract; c, carpel; cr, commissural ring; l, leaf; o, ovule; sam, shoot apical meristem; s, sepal; se, seed; st, stamen; sy, style; v, valve. Scale bars: 50 μm **(A,K)**, 100 μm **(B–D)**, 0.1 mm **(F–I)**, 0.2 mm **(E,J)**.

The expression of *BofrRPL3* is similar to *BofrRPL2* in regard to its early expression in vegetative and floral development. *BofrRPL3* expression is detected during vegetative development in the shoot apical meristem, in the leaf primordia as well as in the procambium and the vascular traces feeding the young leaves. It is also expressed in the adaxial region of more mature leaves (Figure 7A). The expression of *BofrRPL3* during early floral development (stages 3–6) is more similar to the expression found for *BofrRPL2* than to *BofrRPL1*, as it is strongly expressed in the sepal tips, the stamen and carpel primordia and during ovule initiation (Figures 7B–D). Later during flower development, at stage 7, the expression of *BofrRPL3* is strongly maintained in the stamens and in the growing tips of the two carpels that fuse to each other enclosing the ovule (Figure 7E). During carpel development at stages 8–9, *BofrRPL3* is found in the sporogenous tissue of the anthers, toward the adaxial surface of each elongating style, at the tip of the ovule and in the vasculature of the receptacle (Figures 7F–H). *BofrRPL3* is expressed throughout ovule development (Figures 7D–H). During fruit development at stage 10, *BofrRPL3* expression is detected in the 3–4 cell layers of the separation layer between the valves and the commissural ring as well as in the carpel wall (Figures 7I,J). However, this expression is not maintained in the mature fruits at stage 11, where *BofrRPL3* is only restricted to the aril (Figure 7K). In fact, it is the only paralog showing this expression pattern and likely reflects neofunctionalization (Figures 5–7).

### *Papaver somniferum* Carpel and Fruit Development

**FIGURE 8:**
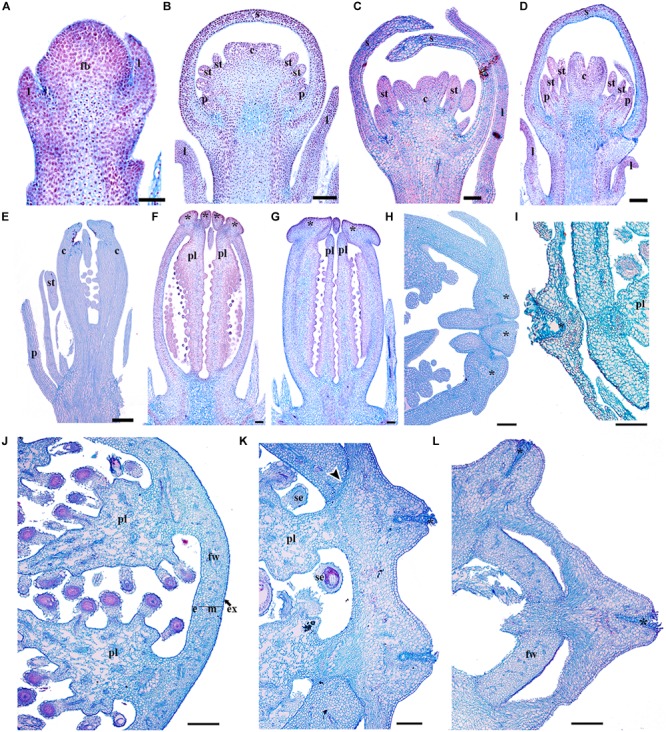
Flower and fruit developmental stages of *Papaver somniferum*. **(A)** Floral bud in stage 1 during sepal initiation. **(B)** Floral bud in stage 3 when petals, stamens and carpel primordia can be distinguished. **(C)** Floral bud in stage 5 with the initiation of a multicarpellate gynoecium and the filament of the stamens. **(D)** Floral buds in stage 6 with the carpels overtopping the multiple ovules. **(E)** Flowers at stage 7 when the initiation of the stigmatic region occurs and the ovules develop by parietal placentation are clearly distinguished. **(F)** Gynoecium in pre-anthesis, stage 8. **(G)** Carpel of a flower in anthesis, the lobules of the stigmas start to elongate. **(H)** Apical region of a young fruit. The tip of the fruit is shown to the right. **(I)** Cross-section of a young fruit showing the apical region with papillose stigma and the fruit wall. **(J)** Cross section through the mid region of a more mature fruit **(K)** Close-up of the crowning stigmatic ring, showing the putative separation layer between the styles and the fruit wall. **(L)** Cross section across the apex of the fruit showing the crowning stigmatic ring. *Asterisks* indicate stigmas, *Black arrowheads* indicate the putative dehiscence region of the fruit, c, carpel; e, endocarp; fb, floral bud; fw, fruit wall; m, mesocarp; p, petal; pl, placenta; s, sepal; se, seed; st, stamen. Scale bars: 50 μm **(A–E)**, 250 μm **(F–H,J–L)**, 500 μm **(I)**.

Our descriptions of the expression analyses for *PsomRPL* in *Papaver somniferum* follow those of Drea et al. (2007) where stages P3, P5, P7, and P8 were defined (hereafter referred to without the P) and Becker et al. (2005), but include a number of previously undescribed developmental stages. We defined floral and fruit developmental stages based on the following developmental landmarks (Supplementary Table S3 and Figure 8). At stage 1, the floral meristem can be distinguished and the sepal primordia are initiating (Figure 8A). Stage 2 is defined by petal initiation. By stage 3 (Drea et al., 2007), stamen and carpel primordia initiate, and the sepals enclose the floral bud (Figure 8B). Stage 4 is defined by the elongation of the multiple carpels. At stage 5 (Drea et al., 2007), the filament of the stamen is distinguishable from the anther and the multiple carpels forming the gynoecium are well differentiated (Figure 8C). At stage 6, the carpels start to fuse (Figure 8D). During stage 7 (Drea et al., 2007), the petals grow and fill the space bounded by the two sepals and in the multicarpellate gynoecium, the ovules initiate on the carpel walls to give parietal placentation, and the stigmatic lobes begin to differentiate (Figure 8E). During stage 8 (Drea et al., 2007), the pedicel of the preanthetic flower undergoes asymmetric growth; the floral buds become pendant right before anthesis and then extend upright when the flower opens in the subsequent stage. Also during stage 8, the fully fused stigmatic tips develop the characteristic upright papillae crowning the distal portion of the gynoecium. The carpel wall is formed by a 11–12 cell layer at this stage (Figure 8F) and remains the same at anthesis corresponding to stage 9 (Figure 8G). Stage 10 is defined by post-fertilization development; the lobes of the stigmas fuse to each other at the tip as a result of residual meristematic activity (Figure 8H), forming a crown-like stigmatic ring (Figure 8I). The fruit wall is formed of 14–18 cell layers, where the exocarp, mesocarp and endocarp are formed of parenchyma cells with small intercellular spaces (Figure 8I). There are multiple vascular bundles in the fruit wall but each carpel has a massive vascular bundle that can be distinguished at the position where the placenta is formed (Figure 8I). At stage 11, the fruit is mature and the fruit wall becomes more compressed with 12–14 cell layers apparent (Figures 8J–L). The apical stigmatic ring is formed of 6–8 cell layers and later during fruit maturation each of the stigmatic rays will produce tension outward antagonizing the poricidal dehiscence zones in each locule (Figures 8K,L). In the poppy variety used in this study, *P. somniferum* cv. Persian White, the fruit does not open leaving the seeds enclosed. Therefore, we will refer here to the dehiscence layer between the fruit wall and the persistent stigmatic ring as a putative separation layer. The putative separation layer is formed of approximately two cell layers between the stigmatic ring and the fruit wall in each of the locules (Figure 8K). Laticifers can be found throughout the pericarp (Figures 8H–L). Fruit dehiscence in wild *P. somniferum* occurs by pores (stage 12), where tension occurs apically below the stigmatic ring and in between the central vascular bundles holding the parietal placenta and the fruit wall in each of the locules leaving only apical pores (Figures 1A,B) (Roth, 1977). The number of pores formed in the pericarp corresponds to the number of locules in the fruit. When the fruit is completely lignified, the rays of stigmas bend outward, these separate along the separation layer between two vascular carpellary central bundles.

### Expression of a *RPL* Homolog in *Papaver somniferum*

**FIGURE 9:**
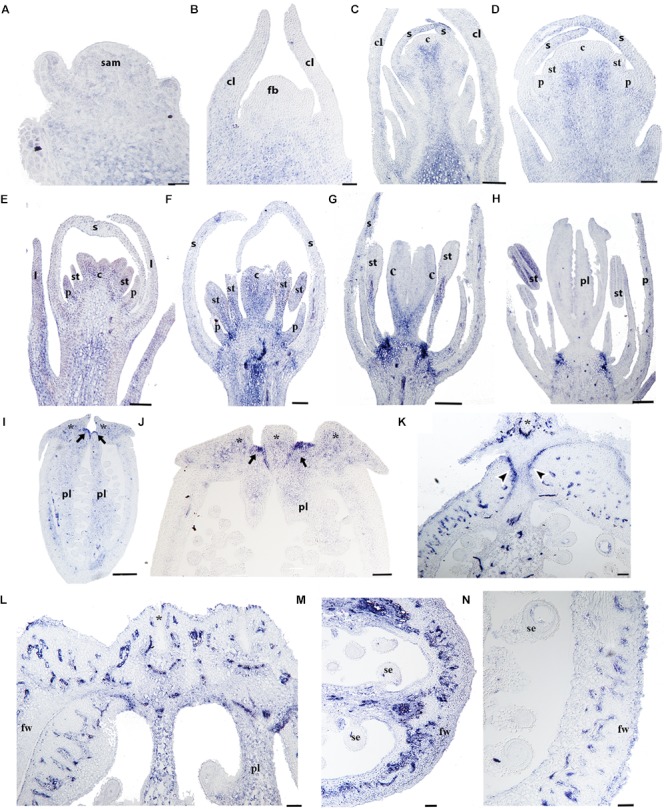
Expression analyses of *PsomRPL* by *in situ* hybridization. Longitudinal **(A–L,N)** and cross-sections **(L–N)** of developing shoots, flowers, and fruits. **(A)** *PsomRPL* expression is detected in the shoot apical meristem, the adaxial region of the leaf primordia and the stem. **(B)** No expression is detected in the floral bud at stage 1. **(C,D)** During stage 3, *PsomRPL* is expressed in the sepal tips. **(E–G)** At floral stage 5–7, *PsomRPL* expression is detected in the petal, stamen, and carpel primordia. **(H)** During pre-anthesis expression becomes restricted to the ovules and on the receptacle in between the fusion of the floral organs. **(I,J)** At stages 9–10, *RPL* is expressed in the style and where the stigmas will form and in the placenta. **(K–N)** In young fruits, *PsomRPL* expression is detected in the vascular bundles, the putative separation layer and the apical region where all the carpels fuse. *Asterisk* stigma, *Black arrows* point to the apical region where the carpels fuse, *Black arrowheads* point to the putative separation layer of the fruit, c, carpel; cl, cauline leaf; fb, floral bud; fw, fruit wall; p, petal; pl, placenta; s, sepal; se, seed; st, stamen. Scale bars: 50 μm **(B–F,K,L–N)**, 100 μm **(A,G),** 200 μm **(H)**, 500 μm **(I)**, 0.2 mm **(J)**.

To better understand the role of *RPL* in Papaveraceae, we analyzed the expression of the single *RPL* homolog identified in *Papaver somniferum* (*PsomRPL*). *PsomRPL* expression is detected during vegetative development in the stem as well as in the shoot apical meristem and the adaxial region of the emerging leaves (Figure 9A). The expression in the floral vascular bundles is maintained throughout floral development between stages 1–9 (Figures 9B–I). *PsomRPL* expression is first detected in the flower at stage 3, where it is found at the tip of the sepals enclosing the floral bud (Figures 9C,D). During stage 5, *PsomRPL* is expressed in the petal primordia, the stamens as well as in the growing tips of the carpel primordia (Figure 9E). At stage 6, *PsomRPL* is expressed in between the floral organs where their proximal portions connect with the receptacle, as well as in the growing petals, stamens and the carpels (Figure 9F). Later during stage 7, the expression in the stamens is restricted to the filament (Figure 9G) as well as to the carpel wall. *PsomRPL* expression is also detected at the junction of each floral organ on the floral receptacle and is maintained during stages 7 and 8 (Figures 9G,H). At stage 8 *PsomRPL* is also expressed in the sporogenous tissue of the anthers and the developing ovules (Figure 9H). At stage 9, *PsomRPL* is differentially expressed in the carpel; it is detected in the region where the carpels fuse, in the extending parietal placentas, and in the endocarp and mesocarp (Figures 9I,J). Later, in the young fruit, *PsomRPL* is expressed in the cells that constitute a putative separation layer between the fruit wall and the stigmas (Figure 9K), in the vascular bundles, the placenta, the epidermis of the fruit wall, and the laticifers (Figures 9L–N).

## Discussion

Very little is known about the fruit developmental network outside the Brassicaceae. *RPL*, particularly, has been described for its function in the proper development of the replum, tissue that is only found in the Brassicaceae fruits (Alvarez and Smyth, 2002; Roeder et al., 2003). Although RPL does not specify replum identity directly, it is a repressor of valve margin and valve identity as specified by *SHATTERPROOF*, *INDEHISCENT* and *FRUITFULL* (Roeder et al., 2003; Girin et al., 2009). In addition, *RPL* has pleiotropic roles in Arabidopsis development including meristem identity, inflorescence, and fruit development as indicated by its many synonyms: BELLRINGER, PENNYWISE, and VAAMANA to name a few (Byrne et al., 2003; Smith and Hake, 2003; Bhatt et al., 2004; Hake et al., 2004). The function of *RPL* orthologs in other angiosperms is only known from the monocot crop species, rice. In rice, it is involved in fruit shedding, and therefore is one of the genes involved in its domestication (Konishi et al., 2006; Arnaud et al., 2011). More recently, expression analyses in selected species of Solanaceae have shown similar expression patterns during flower and fruit development as in Arabidopsis suggesting conserved roles (Ortíz-Ramírez et al., 2018). Expression and functional data available for *RPL* genes are not sufficient to assess their functional evolution across angiosperms. Due to limited functional studies in non-model, non-core eudicots, expression analyses provide a solid base to better predict the functional evolution of this gene lineage. Here, we discuss the expression patterns of *RPL* homologs in two basal eudicots, *Bocconia frutescens* and *Papaver somniferum* (Papaveraceae) whose phylogenetic position is crucial to understanding the functional differences observed between rice (monocots) and core eudicot *RPL* genes; additionally, the two species selected in this study present dry dehiscent fruits with different mechanisms for seed dispersal allowing comparative analyses of *RPL* contribution to opercular and poricidal dehiscence (Kadereit, 1993).

### There Are Two *RPL* Clades Within Ranunculales: *RanRPL1* and *RanRPL2*

According to previous studies *RPL* genes have evolved with the radiation of angiosperms as the result of a duplication event predating angiosperm diversification resulting in the *RPL* clade and its sister clade *POUNDFOOLISH* (*PNF*; Pabón-Mora et al., 2014) (Figure 2). *RPL* are predominantly single copy genes in angiosperms with few exceptions, particularly in taxa corresponding to recent polyploids, such as *Bocconia frutescens, Glycine max, Malus domestica, Papaver bracteatum, Theobroma cacao*, and *Tinospora cordifolia* (Figure 2) and in members of the Solanaceae and Poales, where independent duplications have been found coinciding with known ancient WGD events (Jiao et al., 2011; D’Hont et al., 2012; Pabón-Mora et al., 2014; Ortíz-Ramírez et al., 2018). We performed an exhaustive search in publicly available databases for *RPL* homologs from basal eudicots and we were able to retrieve sequences from all families within Ranunculales. Here, we report an additional duplication, previously unidentified, likely predating the diversification of Ranunculales, resulting in two clades: *RanRPL1* and *RanRPL2* (BS = 94). However, only one gene of *Euptelea pleiosperma* was found nested in the *RanRPL1* clade so it is likely that the duplication event occurred before the radiation of Eupteleaceae but the paralog in the *RanRPL2* clade has not yet been identified (Figure 2). In addition, the two clades vary in terms of the plant groups with representative *RPL* sequences in each. *RanRPL1* only includes sequences of Eupteleaceae, Menispermaceae, and Papaveraceae while *RanRPL2* includes Berberidaceae, Menispermaceae, Papaveraceae, and Ranunculaceae sequences (Figure 2). These differences indicate that some *RPL* sequences were not retrieved even after extensive searches in available transcriptomes (for instance in the case of *Eschscholzia californica*). This can be due to (1) *RPL* gene expression in tissues or organs different from which the transcriptomes were generated in a case by case scenario, (2) low expression of *RPL* copies resulting in low depth and coverage of contigs that remain undetected in the assemblies available (20M), or (3) true gene losses that are harder to assess until more genomes become available. In fact, although we were able to identify two *RPL* copies in *Aquilegia coerulea* (Ranunculaceae), the only basal eudicot with the genome sequenced, no Ranunculaceae sequences were retrieved for the *RanRPL1* clade suggesting that this clade has been lost at least in Ranunculaceae. In addition, these two clades differ in substitution rates and this becomes evident with the long branch formed in the phylogenetic analysis which includes most of the Ranunculaceae and Menispermaceae sequences (BS = 92; Figure 2).

An examination of the conserved domains across basal eudicots (Figure 3), showed the previously identified domains in RPL homologs: the Homeodomain near the C-terminus, (Figure 3B), and the MEINOX INTERACTING DOMAIN (MID) near the N terminus. The MID domain is composed of the SKY and BELL-domains (Hake et al., 2004; Hay and Tsiantis, 2009; Mukherjee et al., 2009). We found that the BELL-domain is conserved in basal eudicots but the SKY motif can be replaced sometimes by SRF (Motif 5, Figures 3A,B) similar to previous results (Pabón-Mora et al., 2014). The location of the ZIBEL motif has been previously found to occur both before the BELL domain and after the Homeodomain (Mukherjee et al., 2009). For the basal eudicot sequences analyzed here, we were able to find the ZIBEL motif (Motif 6 according to the MEME analysis) in some proteins before the BELL domain and in others toward the N terminal region of the protein but never in both locations (Figure 3A) (Pabón-Mora et al., 2014). The MEME analysis allowed us to also identify additional conserved motifs that have not been functionally characterized (Supplementary Figure S2). For example, motif 7 is highly conserved in basal eudicot RPLs and it is located between the BELL and Homeodomain (Figure 3 and Supplementary Figure S2) region, which has been described as highly variable (Mukherjee et al., 2009). Motif 10 is rich in hydrophobic and negatively charged amino-acids and is present only in the *Papaver* proteins included in the analysis: PsomRPL, PabrRPL, and PabrRPL2 (Figure 3A) which may impact protein folding and binding (Nicholls et al., 1991) and therefore confer a specific function to these proteins. In addition, motifs 8, 13, and 20 are found only in the sequences belonging to the RanRPL2 clade but absent in some Menispermaceae and Ranunculaceae proteins such as HycaRPL, NisaRPL1/2/3, XasiRPL1, TicoRPL4 and AqcoRPL (Figure 3A and Supplementary Figure S2). In addition, these proteins are also highly variable toward the N-terminus (Figure 3A).

### *RPL* Expression During Vegetative Development Is Conserved Across Eudicots

To fill the gaps in our understanding of *RPL* evolution across angiosperms and to propose hypotheses in terms of the functional evolution of the *RPL* gene lineage, we analyzed *RPL* expression patterns in *Papaver somniferum* and *Bocconia frutescens* (Papaveraceae; basal eudicots). The two paralogs from *Bocconia* that belong to the *RanRPL2* clade, *BofruRPL2/3*, are expressed in the shoot apical meristem, the adaxial side of the developing leaves as well as in the adaxial side of more developed leaves. The fact that *BofrRPL1* is not expressed in the vegetative tissue suggest some degree of subfunctionalization among the three *B. frutescens* paralogs (Figures 5–7). On the other hand, *PsomRPL*, part of the *RanRPL1* clade, is found to be expressed in the vegetative tissue in shoot apical meristem, developing leaves and in the stem (Figure 9).

*RPL* homologs analyzed here show expression patterns in the vegetative tissue similar to those found in Arabidopsis *RPL* and its sister clade *PNF* (Becker et al., 2002; Roeder et al., 2003; Konishi et al., 2006; Campbell et al., 2008; Arnaud et al., 2011; Pabón-Mora et al., 2014; Bencivenga et al., 2016). Our results suggest that *RPL* function in the maintenance of shoot apical meristem identity, is conserved in monocots and eudicots (Bhatt et al., 2004; Konishi et al., 2006; Kanrar et al., 2008; Rutjens et al., 2009; Arnaud et al., 2011; Mühlhausen et al., 2013). In Arabidopsis, this function is mediated by the interaction with *KNOX* genes (Cole et al., 2006) which also have been found to have a conserved function in Papaveraceae compared to Arabidopsis (Groot et al., 2005). We hypothesize that the meristem identity function mediated by the interaction of *RPL* and *KNOX* genes is likely maintained in eudicots. In fact, its meristematic function may be also maintained in fruit development across eudicots as we found *RPL* expression in *P*. *somniferum* at the apex of the carpel walls were they fuse as result of the post-genital meristematic activity (Figures 8, 9) (reviewed by Girin et al., 2009).

Our results together with those found in Brassicaceae, suggest that the meristematic and vegetative function is shared between the *PNF* and *RPL* clades (Roeder et al., 2003; Smith et al., 2004; Konishi et al., 2006; Kanrar et al., 2008; Ung et al., 2011; Mühlhausen et al., 2013) and that it is likely the ancestral function of these genes in gymnosperm *RPL-PNF* homologs (Pabón-Mora et al., 2014) for which expression or functional studies have not been performed yet. Moreover, it is likely that all BLH proteins are involved in vegetative development, as it is a function that has been broadly described for other paralogs in Arabidopsis (Yu et al., 2009), such as *ARABIDOPSIS THALIANA HOMEOBOX 1* (*ATH1*) that is expressed in the SAM and leaf primordia (Proveniers et al., 2007) and *SAWTOOTH1* (*SAW1 = BLH2*) and *SAW2* (=*BLH5*) that regulate leaf development (Kumar et al., 2007).

### *RPL* Homologs in Papaveraceae Show Broad Expression Patterns During Flower Development and More Restricted Expression During Fruit Development

The floral organ expression patterns of RPL copies in the two Papaveraceae species (Figures 5–7, 9) are consistent with the expression patterns of RPL homologs in Brassicaceae (Roeder et al., 2003; Kanrar et al., 2006; Mühlhausen et al., 2013). Later in the developing carpel, *RPL* is mostly restricted to the stylar and stigmatic adaxial region of *B. frutescens* (Figures 5–7) similar to the expression described in Arabidopsis (Simonini et al., 2018). In general, the orthologs *PsomRPL* and *BofrRPL1*, members of the *RanRPL1* clade are similarly expressed in late stages of carpel development and in the fruit (Figures 5, 9), supporting also some degree of subfunctionalization between the *Bocconia* homologs that belong to the different clades. In Arabidopsis, *RPL* participates in the genetic network involved in the proper development of the style by interacting with auxin response factor *ETTIN* (*ETT*), *INDEHISCENT* (*IND*) and *BREVIPEDICELUS* (*BP*) (Marsch-Martinez et al., 2014; Simonini et al., 2016, 2018). Although *IND* is the result of a Brassicaceae specific duplication event that also gave rise to *HECATE3* genes, pre-duplication *HECATE3-like* genes are likely to maintain the same role in specifying the distal-most portion of the gynoecium (Pfannebecker et al., 2017; Gaillochet et al., 2018). The presence of *BP* orthologs in basal eudicots, known as *KNAT1* (Groot et al., 2005), together with our expression results point to a key conserved role of *RPL* in style development and proper carpel development, as the role of RPL is strictly to repress valve and valve margin developmental genes indirectly inducing replum formation (Alonso-Cantabrana et al., 2007) and in the maintenance of the meristematic activity during plant development, specifically in the fruit (reviewed in Girin et al., 2009).

Of particular interest are the expression patterns of *RPL* in the fruit where dehiscence will occur, whether it is poricidal, in between the carpel central bundles as in *P. somniferum*, or opercular, in between the carpel margins and the commissural ring as in *B. frutescens*. This is suggestive of a role in specifying the separation layer and not the persistent tissue as in Arabidopsis. It is important to notice that even though we used a variety of *P. somniferum* where the fruits do not open, it did not interfere with the *RPL* expression in the putative separation layer of the fruit. The role in replum development may be Arabidopsis specific (Roeder et al., 2003), as it has not been found in other Brassicaceae (Mühlhausen et al., 2013). *B. frutescens* and *A. thaliana* present a similar fruit morphology, with a persistent medial tissue where the seeds remain attached after the two valves fall apart, and some of the genetic mechanisms involved in the fruit development are conserved such as the role of *SPT/ALC* genes in the specification of carpel margins and the dehiscence zone (Groszmann et al., 2011; Zumajo-Cardona et al., 2017) but the proper development of the persistent medial tissue is not determined by the same mechanisms in basal eudicots.

Functional analyses of *RPL* will help us to better understand their contribution to the diversification of fruits within Papaveraceae (Figure 1). Our expression data support the idea that although *RPL* is active during fruit development, its function in the maintenance of a persistent medial tissue is not conserved in basal eudicots. The replum in Arabidopsis seems to be the result of the co-option of *RPL*. During Arabidopsis carpel and fruit development, *RPL* is directly repressed by *APETALA2* (Ripoll et al., 2011) and RPL, negatively regulates *SHATERPROOF1*/2 (*SHP1/2*), which are the paralogs of *AGAMOUS* (*AG*), to the replum boundary (Roeder et al., 2003; Kramer et al., 2004; Zahn et al., 2006; Chávez-Montes et al., 2015). Although no *SHP* orthologs have been found in basal eudicots (Pabón-Mora et al., 2014) it has been suggested that its function in fruit development is maintained by its ancestral gene *paleoAGAMOUS* (Hands et al., 2011). This regulatory network becomes more difficult to understand in *B. frutescens* due to its multiple copies. Nevertheless, the opposite expression patterns of the two copies of *AP2* in *Bocconia* (Zumajo-Cardona et al., 2017) with the expression of the three *RPL* copies presented here, suggest that the interaction of AP2-RPL is also maintained in this species. Thus, based on the protein analysis of *RPL* homologs and the expression patterns related to other genes in the network, it is likely that the interactions between these genes are conserved in Papaveraceae. In addition, we determined that *RPL* in Papaveraceae is expressed in the regions where the carpels distally fuse and subsequently in the separation layer of the fruits. Expression and functional analyses are required in other Ranunculales like Ranunculaceae (i.e., *Aquilegia coerulea*) or Menispermaceae (i.e., *Menispermum canadense*) in order to better understand the role of *RPL* in apocarpous gynoecium as well as the impact of the protein composition as we have detected that *RPL* sequences in these species are highly variable.

Finally, we described for the first time expression of *RPL* homologs in the developing ovules (Figures 5–7, 9). *BELL1*, also a TALE-Homeodomain gene closely related to *RPL* (Becker et al., 2002; Bowman et al., 2016) has been described to function in ovule development. *BELL1* represses *AGAMOUS* (*AG*) in the floral meristem (Bowman et al., 1991a,b; Bao et al., 2004). When *BELL1* is silenced, the continuous expression of AG in the ovule results in homeotic transformation of the integuments into carpels (Modrusan et al., 1994; Ray et al., 1994; Reiser et al., 1995; Hands et al., 2011). Later during ovule formation, *BELL1* is involved in the proper development of the integuments. The expression of *RPL* in the ovules of the two Papaveraceae species compared here (Figures 5–7, 9), particularly in *BofrRPL2* which seems to be specifically expressed in the early formation of the integuments, suggests that *RPL* genes may have the same role in ovule and proper integument development in basal eudicots (Figure 6). Here, we present for the first time also, the expression of *BofrRPL3* in the aril of *B. frutescens* (Figure 7K). Additional expression and functional analyses in non-model species that develop an aril are required in order to determine the conservation of this expression and the role of *RPL* during aril development.

## Author Contributions

All authors planned and designed the research, performed the experiments, analyzed the data, and wrote the final version of the manuscript. All authors read and approved the final manuscript.

## Conflict of Interest Statement

The authors declare that the research was conducted in the absence of any commercial or financial relationships that could be construed as a potential conflict of interest.

## Footnotes

1 https://sites.google.com/a/ualberta.ca/onekp/

2 https://bioinformatics.tugraz.at/phytometasyn/

3 https://mafft.cbrc.jp/alignment/server/

4 http://tree.bio.ed.ac.uk/software/figtree/

5 http://meme-suite.org/

## Supplementary Material

FIGURE S1 BofrRPL1, 2, 3 and PsomRPL protein sequences showing the regions where specific primers were designed. BofrRPL reverse primers were designed on the 3′UTR.

FIGURE S2 MEME analysis showing conserved motifs across basal eudicots RPL protein sequences. *Letter size* denotes the degree of conservation of each amino acid.

TABLE S1 List of the genes included in the phylogenetic analysis, with corresponding species, family and accession number of the sequences.

TABLE S2 Primers used for the *in situ* hybridization analyses in the four RPL homologs.

TABLE S3 Developmental landmarks for each stage of flower and fruit development in *Papaver somniferum*, based on Drea et al. (2007) and our complementary stages.
