# Supplementary figures and images for: Duplication and Diversification of REPLUMLESS – A Case Study in the Papaveraceae
Source: Front Plant Sci. 2018 Dec 12;9:1833. doi: 10.3389/fpls.2018.01833 (PMC6299025; doi:10.3389/fpls.2018.01833)

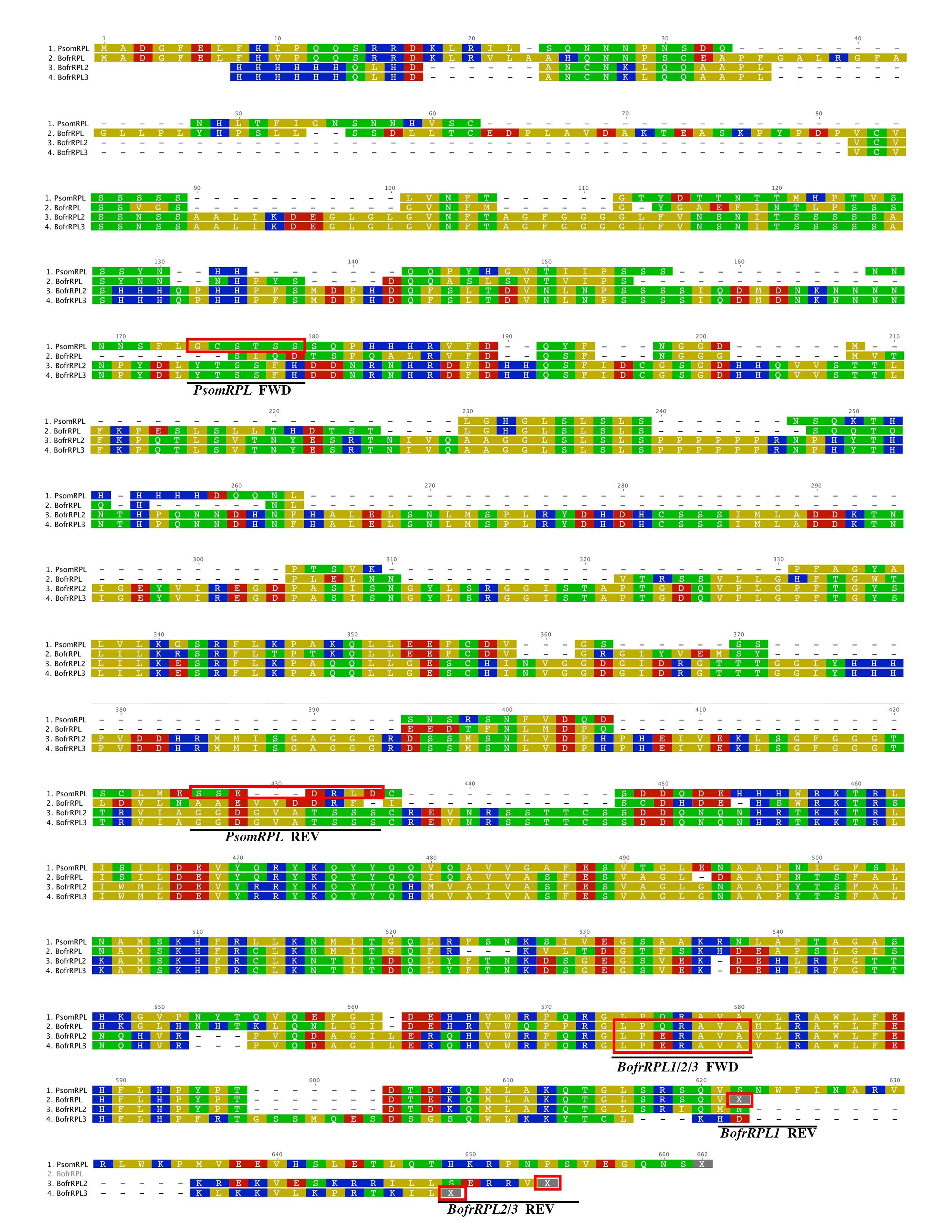

Supplement: FIGURE S1 — BofrRPL1, 2, 3 and PsomRPL protein sequences showing the regions where specific primers were designed. BofrRPL reverse primers were designed on the 3′UTR. [file Image_1.TIF]

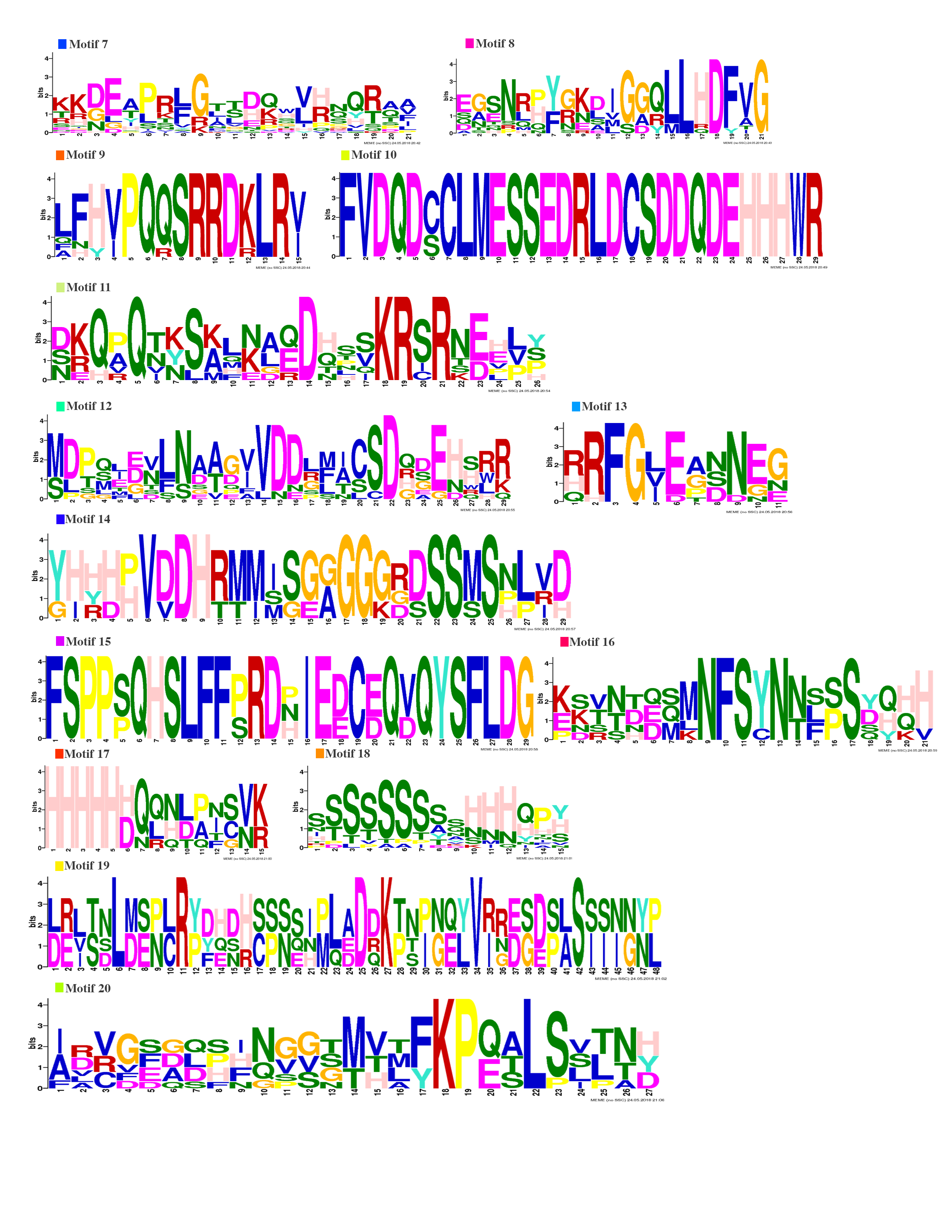

Supplement: FIGURE S2 — MEME analysis showing conserved motifs across basal eudicots RPL protein sequences. Letter size denotes the degree of conservation of each amino acid. [file Image_2.TIF]
